# Renegotiating dimensions of the self: A systematic review and qualitative evidence synthesis of the lived experience of self‐managing rheumatoid arthritis

**DOI:** 10.1111/hex.13122

**Published:** 2020-09-01

**Authors:** Susie Donnelly, Molly Manning, Hasheem Mannan, Anthony G. Wilson, Thilo Kroll

**Affiliations:** ^1^ Centre for Interdisciplinary Research Education and Innovation in Health Systems (IRIS) School of Nursing, Midwifery and Health Systems University College Dublin Dublin Ireland; ^2^ School of Allied Health Faculty of Education and Health Sciences University of Limerick Limerick Ireland; ^3^ Department of Social Sciences School of Liberal Education FLAME University Pune India; ^4^ Centre for Arthritis Research School of Medicine and Medical Science Conway Institute University College Dublin Dublin Ireland

**Keywords:** lived experience, meta‐synthesis, qualitative evidence synthesis, rheumatoid arthritis, self‐management, systematic review

## Abstract

**Background:**

As chronic illnesses, such as rheumatoid arthritis (RA), place an increased burden on health‐care systems, the ability of individuals to self‐manage these diseases is crucial.

**Objective:**

To identify and synthesize the lived experience of self‐management described by adults living with RA.

**Design:**

A systematic search of five electronic databases (MEDLINE, CINAHL, EMBASE, PsycINFO and ASSIA) was undertaken to identify relevant studies. Data were extracted and quality‐assessed using CASP guidelines. A meta‐synthesis was conducted based on Thomas and Harden's thematic synthesis approach.

**Results:**

The search identified 8423 publications. After removing duplicates, 6527 records remained of which 32 studies met the inclusion criteria. Quality of studies was moderate to high, yet a considerable lack of reflection on researcher bias was evident. Our analysis identified 28 dimensions of self‐management RA across six domains: (a) cognitive‐emotional, (b) behavioural, (c) social, (d) environmental, (e) physical and (f) technological. Cognitive‐emotional experiences dominated the analysis. Renegotiating ‘the self’ (self‐concept, self‐esteem, self‐efficacy) was a key focus of self‐management among individuals with RA.

**Conclusion:**

Our findings highlight the focus of ‘the self’ as a central concern in the self‐management of RA. Standardized self‐management programmes may primarily focus on disease management and daily functioning. However, we suggest that personal biographies and circumstances should move to the fore of self‐management support.

**Registration:**

PROSPERO International Prospective Register of Systematic Reviews 2018: CRD42018100450.

**Patient or Public Contribution:**

Patient and public involvement was not explicit in this review. However, three authors provided a patient perspective on the self‐management of arthritis and autoimmune disease.

## 1 INTRODUCTION

Rheumatoid arthritis (RA) is a highly prevalent chronic autoimmune disease affecting between 0.3% and 1% of the global population. It is more prevalent in developed countries and among women. It is characterized by inflammation of the joints causing pain, stiffness and swelling. It can affect all areas of life, including work,^1^, ^2^ family^3^ and leisure activities,^4^ as well as physical and mental health.^5^ Symptoms tend to emerge between the ages of 20 and 40, and within ten years, at least 50% of patients in developed countries are unable to maintain full‐time employment.^6^ The term ‘self‐management’ refers to the ‘day‐to‐day tasks an individual must undertake to control or reduce the impact of disease on physical health status’.^7^, ^8^ It involves the patient taking increased responsibility for their health in terms of the decisions they make and activities they engage in and can greatly improve health outcomes and the individual's quality of life.^9^ Self‐management may require individuals and their families to make extensive practical as well as psychosocial adjustments. For example, individuals may find they are unable to perform previously taken‐for‐granted behaviours, routine tasks and activities, and require greater practical and emotional support from others.^10^ Patient education is recognized as integral to the success of self‐managing a chronic illness, and great strides have been made in the development and implementation of Chronic Disease Self‐Management Programmes (CDSMP).^11^, ^12^, ^13^, ^14^ However, despite advances in patient education programmes and biologic treatments, adherence to long‐term therapies remains a challenge.^15^, ^16^, ^17^, ^18^ Non‐pharmacological treatment, such as exercise, diet and lifestyle modifications are particularly difficult behaviours to adopt.^19^ Moreover, effective supports and interventions might not be suitable or accessible to everyone.^11^ For example, people from low socioeconomic and disadvantaged groups report poorer outcomes and lower levels of adherence in CDSMPs.^20^, ^21^ In turn, this may exacerbate the social gradient in chronic disease outcomes.^22^ Thus, consolidating qualitative evidence around the lived experience of self‐managing RA could inform the development of self‐management support and resources.

A recent *mega*‐ethnography^23^ summarized evidence from nine qualitative evidence syntheses exploring experiences of RA and established that living life with RA is a precarious and marginalizing experience, which includes RA as an emotional challenge, as an invisible illness and presenting a biographic disruption.^23^ While there is much qualitative research exploring the lived experience of RA in recent years, to date, to the best of our knowledge there is no qualitative evidence synthesis addressing the lived experience of self‐management. The purpose of this paper was to systematically identify, appraise and synthesize prior qualitative studies of RA to examine the lived experience of self‐management. We seek to answer the following research questions: (a) How do patients experience the self‐management of RA? and (b) What aspects of their experience are described as most pertinent to living with and managing the condition?

## 2 METHODS

This study presents a systematic review and synthesis of qualitative evidence of the lived experience of self‐managing rheumatoid arthritis. Our approach involves a comprehensive search for and retrieval of qualitative research publications, a critical appraisal of primary studies, a classification of results and a synthesis of key findings.^24^ This work was conducted following the ‘Enhancing Transparency in Reporting the Synthesis of Qualitative Research (ENTREQ)’ guidelines, and a copy of the ENTREQ checklist is available in Appendix E.

### 2.1 Search strategy

A highly sensitive search strategy was constructed with guidance from a university health sciences librarian and consisted of terms pertinent to ‘lived experience’, ‘rheumatoid arthritis’, ‘self‐management’ and ‘qualitative research’ (Appendix A). Five indexing databases spanning life sciences and biomedicine (MEDLINE, EMBASE), nursing (CINAHL) and social sciences (PsycINFO, ASSIA) were searched in March 2018 and again in January 2019. Diverse databases were selected to ensure the search was comprehensive. This was supplemented by hand‐searching reference lists and bibliographies of retrieved publications.

The inclusion criteria for the articles were organized following the SPIDER framework for qualitative literature^25^: *sample:* adults, aged 18+ with a diagnosis of RA; *phenomenon of interest*: lived experience of adults with RA with self‐management; and *design/ evaluation/ research type*: qualitative peer‐reviewed primary studies of any design in English language. There was no limit on the year of publication. Mixed‐methods studies and studies examining the perspectives of mixed participant groups were included only if data pertaining to participants with RA could be isolated. We included primary qualitative data obtained and interpreted using qualitative methods of data collection (including interviews, focus groups and ethnography) and analysis. We excluded non‐empirical articles including opinion pieces, commentaries, systematic reviews and grey literature. We included only peer‐reviewed journal articles to maximize the methodological quality and reporting of the included studies.^26^ A full list of inclusion and exclusion criterion is included (Appendix B).

### 2.2 Study selection

Search results were downloaded to reference management software (EndNote version 11), and duplicates were removed. Citations were checked for eligibility in two stages, and screening decisions were informed by a standardized instrument prepared by the first author in consultation with the team (Appendix B). Firstly, titles and abstracts of records were screened independently by two authors [SD & MM]. Secondly, full‐text screening was conducted by one author [SD] with a purposive sample of 10 full‐text articles screened by a second author [HM]. At each stage, discrepancies were resolved through discussion with a senior member of the team [TK].

### 2.3 Critical appraisal

All included publications were subject to a global assessment of study quality using the Critical Appraisal Skills Programme (CASP). This assessment was conducted by the first author in consultation with the team. It involved a systematic assessment of each component attributing a score to indicate whether the item had been adequately addressed. The studies were scored out of 10. Scores >9 were deemed high quality; scores between 7 and 9 were deemed moderate quality; and scores <7 were low quality. The ‘typology’ and characteristics of the qualitative evidence (Appendix D) were also assessed and reported.^27^

### 2.4 Data extraction

All text under the headings ‘results’ was extracted electronically and entered into computer software (NVivo version 11) by the first author [SD]. The remaining sections, such as the conclusions, were scanned for any additional findings and extracted where relevant.

### 2.5 Data synthesis

Drawing from both meta‐ethnography and grounded theory, Thomas and Harden's^28^ 'thematic synthesis' approach was used to synthesize the extracted data. Our intention was not to simply aggregate findings, rather we aimed to produce descriptive and analytical themes by translating concepts from individual studies into one another resulting in the development of overarching concepts and new insights. The process of deriving themes from the data was inductive. [SD] performed a close reading of all papers. In the first stage of coding, central categories were identified [SD] and [TK]. These were conceptualized as domains within which self‐management was experienced. The second stage of analysis involved line‐by‐line coding of the data to identify dimensions of people's experience and assess how they fit within existing domains, or whether new domains needed to be identified. This iterative process involved comparison and reflection between and within codes. Analysis was led by [SD]*,* while [TK], [MM] and [HM] contributed to its refinement at various stages.

## 3 RESULTS

### 3.1 Study selection

**Figure 1:**
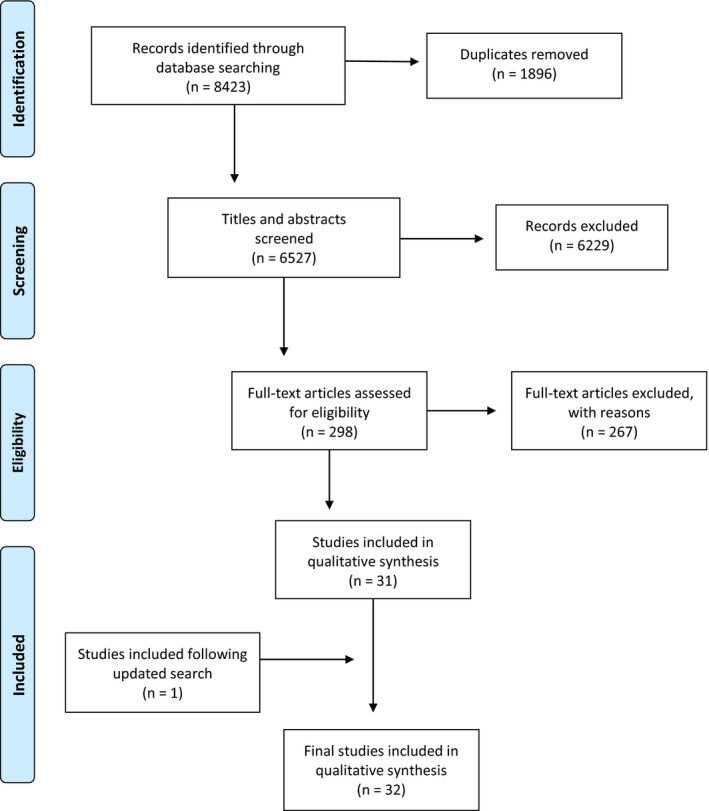
PRISMA flow chart of literature search process and inclusion of studies

The electronic database search identified 8423 citations. After removing duplicates, 6527 records remained. Based on this screening, 298 records were deemed potentially eligible for inclusion and full texts were accessed for further scrutiny. Following full‐text screening, 31 studies were deemed to have met the selection criteria. An updated search in January 2019 resulted in the inclusion of one additional study. Thus, a total of 32 studies were ultimately identified for analysis. The process is reported in Figure 1 using the Preferred Reporting Items for Systematic Reviews and Meta‐Analyses (PRISMA) statement.^29^

#### 3.1.1 Characteristics of included studies

The 32 studies included in this meta‐analysis describe the lived experience of approximately 665 people with rheumatoid arthritis. Most studies (n = 640) sampled people with rheumatoid arthritis. Except two studies which included mixed disease populations, incorporating people with psoriatic arthritis (n = 6), unspecified polyarthritis (n = 2)^30^ and juvenile idiopathic arthritis (n = 2).^31^ In some cases, samples were drawn from larger studies (eg clinical trials) and there may be duplication of participants as a result.^32^, ^33^, ^34^, ^35^, ^36^ Comorbidity was discussed in one study but only to highlight it as an exclusionary criteria.^37^ Apart from an Iranian study, all participants were from developed countries, predominantly within North America or Europe. The publication period ranged from 1993 to 2018. The disciplinary affiliation of lead authors was predominantly clinical (nursing n = 9; medicine n = 7; physio/occupational therapy n = 4; public health n = 3), while social scientists represented a minority (health sciences n = 6; social work n = 2). In terms of research design, the studies referred to using a phenomenological approach, grounded theory or ethnography and employed some variation of content or thematic analysis. In nine of the studies, the sampling approach was unspecified. Where sampling was stated, purposeful sampling was most often used. Most studies (n = 28) used semi‐structured interviews as the main instrument for data collection, sometimes referred to as ‘open‐ended’ or ‘conversational’ interviews. In four studies, focus groups were used. Locations of data collection varied from a hospital or similar clinical site, such as an office or private room at a rheumatology clinic or health centre (n = 9); in seven studies, interviews were conducted at non‐clinical sites only (eg at the participants home, workplace or university), while a further seven stated that interviews were carried out at a mix of clinical and non‐clinical sites. In one study, interviews were conducted by telephone. Eight studies did not specify a setting or location of interviews. A comprehensive breakdown of the characteristics of included studies is included in Appendix D.

### 3.2 Quality of included studies

Using the CASP assessment tool, we determined that 17 of the studies were high quality; 15 were moderate quality; and none were considered low quality. All studies provided a clear statement of aims and findings and used appropriate qualitative methodologies. Some weaknesses were revealed in terms of rigour, specifically limited consideration of the relationship between researcher and participant (eg a critical examination of the researcher's own role and potential bias). In 14 studies, the researcher failed to critically examine their own role, potential bias and influence. In terms of credibility, it was sometimes unclear whether ethical issues had been taken into consideration. While researchers tended to state that ethical approval had been granted by a relevant institutional committee, it was observed that meaningful engagement with ethical issues was unclear in 16 studies. In seven studies, the value of the research was not addressed. Recognizing the limitations of applying quality appraisal tools to produce consistent judgements, and to avoid the risk of overlooking more insightful studies, publications were not excluded based on the CASP assessment.^38^, ^39^, ^40^ However, the process of systematically assessing and characterizing the literature served to highlight overall strengths and weaknesses in our sample (Appendix C).

### 3.3 Synthesis

From a meta‐synthesis of 32 studies, we identified six conceptual domains in which the self‐management of RA was experienced: (a) cognitive‐emotional; (b) behavioural; (c) social; (d) environmental; (e) physical; and (f) technological. Table 1 summarizes 28 dimensions of the lived experience of self‐managing rheumatoid arthritis across five domains providing illustrative quotes.

**Table 1:** Summary of conceptual domains and dimensions of the lived experience of self‐managing rheumatoid arthritis

Domain of experience		Dimensions	Illustrative quotations
(i) Cognitive‐emotional (what we think and feel)	1	*Acceptance,* *lack of*	‘I always thought I’d wake up [the] next morning [and] it'd be gone. But that never happens and so you do learn to accept it in the long run and I think I handle it better now … by knowing m[y] limitations’.^29^ ^(p200)^ ‘I ignore it, basically. Try and get on with day‐to‐day things… I bury my head in the sand. I don't…even bother treating the symptoms’.^40^ ^(p209)^
	2	*Anger, frustration, irritability*	‘I use [running] as a way of getting rid of the frustration and the anger; I just run and run and run until I can't run anymore…I just feel like just punishing myself. I know I shouldn't because I know I’m going to pay for it in the long run’.^41^ ^(p334)^ ‘When you feeling in pain and you're aching and you're tired, you haven't got a lot of patience with the children which really gets me down'cos I think, you know, I shouldn't be shouting at them it's not their fault … Everything you do, you know, just normal household duties [and] looking after a baby is really hard work’.^42^ ^(p201)^
	3	*Blame, cause*	‘It just seems random… well, to me… to me it just seems random’.^40^ ^(p207)^ ‘In my opinion, stress is the main reason why my fl are comes about. Every time I stress about things, it just sets off’.^40^ ^(p207)^
	4	*Social comparison*	‘Now, compared with the other ladies who had deformities in their hands, I was in a better condition…When I see people who are in a worse condition, I'm thankful to God that I am still not at that stage’.^14^ ^(p246)^ ‘Everybody around me enjoys their healthy and happy life, except only me. I never do [a] bad thing to anybody. I always try to do good for everybody in [my] whole my life. Why does the God give me such pain? It's unfair’.^43^ ^(p242)^
	5	*Depression, sadness, despair, suicide*	‘It's a vicious cycle with RA. You feel depressed, so your arthritis acts up more, and the more it acts up, the more depressed you get, until you break it somehow’.^14^ ^(p294)^ ‘I would have taken my own life. Lousy, I couldn't get dressed, nor even could I cover with the sheets. It is a very bad pain. Living like that is not worth it’.^44^ ^(p07)^
	6	*Hopes and fears*	‘Yes, I’m thinking of a woman, a woman in a wheelchair who cannot move and is totally handicapped. I hope this won't happen to me. And then I think about the future. When it starts like this, how will it continue?’^34^ ^(p68)^ ‘Perhaps I can slow it [RA] up…get it to stabilise’^45^ ^(p239)^
	7	*Optimism, positivity, humour*	‘What has been most helpful to me (laughter) a sense of humor’^37^ ^(p512)^ ‘I think, I have begun to think really positively, that I will get better’.^34^ ^(p68)^
	8	*Religion and spirituality*	‘I go to church every day to pray to God to recover from the disease’.^43^ ^(p243)^ ‘…I believe in God and it's that what keeps me going, so I don't worry. God's got the best for me; I met my husband through God’.^46^ ^(p197)^
	9	*Self (concept, esteem, efficacy)*	‘One feels one is worth less compared to before…’.^32^ ^(p34)^ ‘But I can't stop and live… I have to overcome so to speak…through building up in some way a confidence in oneself, that you can fix this. I think that you can be quite capable if you focus and most things are able to be solved with a little thought’.^47^ ^(p97)^
	10	*Shame, guilt, embarrassment*	‘I can't even open up a bottle of mineral water on the plane, it's embarrassing. So I got to call for the air stewardess to help me to open it up. People think that I am trying to get fresh’.^48^ ^(p383)^ ‘I feel guilty for not being able to be with them (daughters), neither physically nor emotionally’.^44^ ^(p9)^
(ii) Behavioural (what we do; action taken)	11	*Adjustment and adaptation*	I think even when I’ve had a swollen knee I’ve got on the bike and just pedalled more with one leg than the other ^49^ ^(p699)^ ‘The person with this old body of mine hasn't recognized that he has to adjust, he hasn't got time for it…’.^45^ ^(p32)^
	12	*Planning, pacing*	‘I knew that I’d be doing a lot of walking so I made sure that the next day was empty’.^28^ ^(p700)^ ‘It is a bit like planning your life. It is like this motto for the day, to plan your day, plan what you are going to do. It is like that, you always have to base your occupations on how the day is and what you feel like doing, what you can do’.^33^ ^(p87)^
	13	*Self‐care*	‘I want to manage on my own, even if it takes more time. To be able to take care of your personal hygiene and to get dressed of your own accord is number one, so that I can be independent of others’.^31^ ^(p85)^ ‘…now I am working on becoming relaxed and loosening, and it is not easy for me. But I work a lot on it. Maybe I am behaving too modern now: I take belly dancing classes now, in order to relax physically as well as mentally’.^50^ ^(p663)^
	14	*Help‐seeking*	‘It's always the last port of call coming to see the rheumatologist. I go through absolutely everything at home before I come and see them’.^49^ ^(p701)^ ‘I have to kind of accept it and just do the best that I can with it [RA]. Learn to ask for help which I’m not very good at doing that. Where I wouldn't ask people for help, now I do’.^37^ ^(pp511‐512)^
(iii) Social (interaction with others and roles)	15	*Invisible illness*	‘It's almost like, I’d like a visib[le] sign of my disability… in other words I’d like a white stick or I like a wheelchair, because of my age and my look, people don't see me as disabled, why should they?’.^51^ ^(p115)^ ‘When they come [the children], I pretend I’m not doing so bad. I make myself up, I dress up, for they wouldn't notice’.^44^ ^(p9)^
	16	*Domestic roles*	‘As a man you should be the main person doing the lawns, the gardens, round the house’.^41^ ^(p333)^ ‘Everybody helps out, I don't have a choice. I don't think my kids miss out; my little one may be, because I can't go out with her, swimming for example. But my sister and husband and other family members take them out. My husband does a lot of the housework now. He does work hard to keep things going’.^51^ ^(p112)^
	17	*Employment*	I mean I worked for nearly 40 years, there is a certain loss of identity when you can't work anymore… When you can't work, not only have you taken away your sort of daily structure, you have taken away a large part of your social life ^51^ ^(p115)^ ‘Why don´t you work in the profession you are qualified for? Oh, that's because I have a joint disease. I really don´t bother to explain. They don´t understand anyway’.^32^ ^(p34)^
	18	*Economic*	‘Using a swimming pool has become very expensive. Before, it only cost 10,000 Tomans per session. Now, if you add the transportation cost, you have to spend about 25,000 Tomans for a session. I cannot afford it’.^52^ ^(p245)^ ‘…the first line of treatment is a few hundred dollars a month. When you're deeper into your disease it's $1,500 a month. A lot of people are not aware’.^48^ ^(p386)^
	19	*Gender*	‘It puts you in a position where you have to ask for help and it's not a very sort of macho thing’.^30^ ^(p332)^ ‘I had to cut my hair off, my hair used to be long… I couldn't manage it, so it has to be short, so it doesn't need blow drying because I can't get my arm above my head. Yeah, this type of practical things take[s] away some of your femininity, or change[s] who you are’.^51^ ^(p115)^
	20	*Loss, loneliness, isolation*	‘I live on my own…so I can rattle around, chuck stuff and just be thoroughly frustrated and just lay in bed all day’.^41^ ^(p334)^ ‘I used to invite people over very often. These days, since I cannot take care of my guests the way that I used to, I don't like to have guests at my house anymore’.^51^ ^(p244)^
	21	*Personal and social relationships*	My husband helps me but I know deep down that he doesn't get any joy from me. I don't have the energy to be with him. Got no appetite, nothing. I’ve lost my appetite for life! ^53^ ^(p2651)^ ‘Because of their attitude, I don't socialize with my relatives. They annoy me. For example, I go to visit them for an hour and they start asking me about my illness and comment about my life. I can't take it. No one will tolerate such behaviours’.^52^ ^(p5)^
(iv) Environmental (setting in which we manage)	22	*Access and built environment*	‘It is really difficult for me. I live [on] a higher floor of a building and have to climb two flights of stairs every time I want to go somewhere’.^52^ ^(p245)^ ‘I can't reach anything on my desk any more. Everything [must be] within 12 or 15 inches of the front of my desk’.^54^ ^(p228)^
	23	*Weather and temperature*	‘In the winter, I cannot come here [the clinic] by myself; someone has to bring me. If I want to come by myself using public transportation, then I have to change buses. Exposure to cold weather is not good for me; it makes me sick’.^52^ ^(p245)^ [RA is] ‘like a weather forecast. If it's going to rain, I get a bit of pain; if we get snow or frost, I get quite a bit of pain’.^55^ ^(p207)^
(v) Physical (the body in which we manage)	24	*Body as ill, deformed, disabled*	‘My body is changing so strangely’.^43^ ^(p242)^ ‘What bothers me most are my deformed hands, and being self‐conscious is worse than the pain, but my hands are something I have to live with, and by the grace of God, I keep going’.^56^ ^(p284)^
	25	*Symptoms*	‘By the end of the workday, I was crying in excruciating pain while scooting up and down the stairs at home, and it took almost a year before I felt somewhat normal’.^56^ ^(p284)^ ‘I do suffer quite a lot from fatigue, so it really depends how much energy I have to do that and then also if something's quite sore I might not want to, you know, I might cancel a shopping trip if I’ve got a very sore knee or ankle or something, knowing that it's going to aggravate it ’.^49^ ^(p700)^
(vi) Technological (technologies used to manage)	26	*Assistive devices and aids*	‘I was such an opponent in the beginning, not wanting to have a wheelchair or a [powered wheelchair]…but now I couldn't be without it, it's changed my life a lot… When it's really difficult I just take my [powered wheelchair] and go out, just enjoying it, meeting people and disengaging my pain’.^53^ ^(p2651)^ ‘Assistive devices are important in the household, for instance. Really important! I have got the full kit since the 80s. I can't cut things at somebody else's place, so I have to bring my own stuff or, you know, a nutcracker on all my journeys to be able to open bottles, for instance’.^31^ ^(p87)^
	27	*Health‐care professionals and services*	‘[The HCP] didn't have any specific recommendations for me. Only if I asked a question, they answered it. Nothing more’.^52^ ^(p245)^ ‘I still lean on my doctor telling me it's going to be alright’.^32^ ^(p35)^
	28	*Medical treatment*	‘When I started with the medication, I saw the light in the tunnel…Now I’ll be cured… Now I’m ready, now it's good! And then I get it into my head and set my ambitions very high. But it vanished ‐ because it didn't turn out as I thought it would’.^53^ ^(p2650)^ ‘I started to try natural things and there was a time I completely stopped the prescribed medication’.^44^ ^(p11)^

#### 3.3.1 Cognitive‐emotional

All studies contributed to the cognitive‐emotional domain. This refers to participants’ thoughts and feelings about living with and managing RA. Learning to accept the illness was an important part of managing life with RA.^31^, ^32^, ^33^, ^34^, ^37^, ^41^, ^42^, ^43^, ^44^, ^45^, ^46^, ^47^, ^48^, ^49^, ^50^, ^51^, ^52^, ^53^, ^54^, ^55^ This involved accepting the pain associated with the condition, recognizing one's limitations and adapting one's behaviour accordingly. Social comparison was a common strategy across the studies.^30^, ^32^, ^34^, ^41^, ^44^, ^45^, ^46^, ^48^, ^49^, ^51^, ^53^, ^55^, ^56^, ^57^, ^58^, ^59^ Downward comparisons were used to compare oneself to someone in a worse situation or condition: *‘…I can at least think and talk and decide what I want to do today and what I can't do today…Yes, I’m still lucky because a lot of people are worse off'*.^41^ ^(p2651)^ This could be a helpful to recognize one's physical and mental improvements, for example as a result of successful medical treatment or increased resilience. Upward comparisons were made with those perceived as ‘better off’: ‘*Even patients with leprosy are more scared of rheumatoid arthritis than their disease. It is a worst punishment of God in the world’*.^46^ ^(p242)^ Self‐comparison, also described as ‘temporal comparison’,^45^ was a feature of people's experience whereby the person made comparisons with how they were prior to the onset of RA. This could manifest upwardly resulting in a feeling of being less physically able than before the onset of the illness or downward: ‘*Now, I see life in a whole different light, [I] try not to project anything negative, take one day at a time, count my blessings, and it's been a good lesson for me’*.^57^ ^(p282)^.

As part of the emotional work of self‐management, individuals were required to deal with feelings of frustration, anger and depression. Anger was a prominent emotional response^34^, ^43^, ^44^, ^45^, ^46^, ^48^, ^49^, ^52^, ^53^, ^57^, ^58^, ^59^, ^60^ and formed part of the burden of coping with RA. This was often rooted in frustrations around limitations imposed by pain and fatigue. Participants described a sense of injustice towards developing the illness. This created a need to identify a cause.^36^, ^46^, ^47^, ^54^, ^55^, ^60^, ^61^ Feelings of sadness, despair and depression were common.^34^, ^48^, ^49^, ^51^, ^53^, ^57^, ^58^, ^59^, ^60^, ^62^ In some instances, participants expressed suicidal ideation. Hopes and fears were often addressed.^30^, ^32^, ^33^, ^34^, ^35^, ^36^, ^37^, ^41^, ^42^, ^44^, ^45^, ^46^, ^48^, ^49^, ^51^, ^52^, ^55^, ^56^, ^57^, ^58^, ^59^, ^60^, ^63^ Participants were concerned how they would manage their illness in the future. Despite advances in the treatment, participants managed their illness under the perceived threat of immobilization, deformity and dependence. To counter this, strategies of optimism, positivity and humour were frequently adopted.^30^, ^31^, ^33^, ^34^, ^35^, ^37^, ^42^, ^46^, ^49^, ^53^, ^54^, ^58^, ^59^ A small number of studies described how religious belief and practice provided hope and helped to maintain a positive outlook.^46^, ^47^, ^48^, ^56^, ^57^, ^62^

The predominant feature of the lived experience of self‐managing RA related to ‘the self’, a broad category constructed to reflect various dimensions of self‐concept (beliefs about oneself), self‐esteem (self‐worth and value) and self‐efficacy (confidence in ability to deal with health problems). These concepts were found in 29 of the 32 studies.^31^, ^32^, ^33^, ^34^, ^35^, ^37^, ^41^, ^42^, ^43^, ^44^, ^45^, ^46^, ^47^, ^48^, ^49^, ^50^, ^51^, ^52^, ^53^, ^54^, ^55^, ^56^, ^57^, ^58^, ^59^, ^60^, ^61^, ^62^, ^64^ RA was often experienced as a threat—or disruption—to self‐identify. How an individual perceived themselves as ill (or not) was intrinsic to how they approached self‐management and the role it played in their lives.

Physical deformity played a role in relation to self‐identity, self‐esteem and self‐confidence. Concealing deformity was important to some participants, especially in work and social situations: *‘In a couple's party, I have to [be] extremely careful to conceal my deformed appearance. It's very hard for me, but I have to do it to keep my self‐esteem’*.^46^ ^(p243)^ Masking and concealing illness from others could produce negative feelings: ‘*Everything should be as perfect as possible, so that you couldn't see that I was sick… this meant that I almost cracked up and got depressed…’*.^32^ ^(p32)^ Feelings of shame, embarrassment and guilt around various aspects of self‐managing the condition were described.^32^, ^34^, ^36^, ^50^, ^51^, ^52^, ^55^, ^56^, ^59^ These feelings were expressed, for example, in relation to using visible aids, guilt around not being physically or emotionally available to others, and feelings of embarrassment around asking for help.

#### 3.3.2 Behavioural

Behaviour relates to what the person does; it is an act or an action taken. Across the 32 papers analysed, the central idea that appeared in relation to behaviour concerned adjusting and adapting to RA.^30^, ^31^, ^32^, ^33^, ^34^, ^35^, ^37^, ^41^, ^42^, ^44^, ^45^, ^46^, ^47^, ^48^, ^49^, ^50^, ^53^, ^54^, ^55^, ^57^, ^58^, ^62^ People with RA described how they altered their approach to daily tasks and activities in line with reduced strength and functioning. As Ostlund found, ‘*behavioural adjustments included more rest, such as going to bed earlier or taking a nap during or after work, in order to be able to fulfil their duties’*.^50^ ^(p210)^ Adjusting and adapting to an illness required resilience and the ability to draw from accumulated knowledge to employ effective self‐management behaviours.

Pacing and planning daily activities commonly featured in individual's experiences.^30^, ^31^, ^34^, ^35^, ^36^, ^37^, ^41^, ^42^, ^43^, ^44^, ^45^, ^48^, ^49^, ^50^, ^51^, ^52^, ^53^, ^55^, ^57^, ^58^, ^59^, ^60^, ^61^, ^62^, ^63^ Participants described the need to be selective in the activities they chose to engage with. There was a sense of trading‐off and ‘dosing’ certain activities. Lutze observed that participants ‘took the risk of stretching their limits because they so dearly wanted to do special things, or to just feel the pleasure of being "normal"’.^34^ ^(p66)^ People with RA reported a need to carefully plan their lives in a way that minimized impact on their joints, preserved their energy and avoided additional pain and fatigue. Consequently, self‐management reduced spontaneity and created a cognitive burden. Added to this was the unpredictability of symptom flares. The lack of a clear cause of a flare created a sense of uncertainty and reduced self‐efficacy.

Physical symptoms of RA created difficulties in managing aspects of self‐care, such as exercise and diet, self‐maintenance and hygiene (ie getting dressed or brushing hair/ teeth).^31^, ^34^, ^35^, ^36^, ^41^, ^46^, ^47^, ^49^, ^51^, ^53^, ^54^, ^55^, ^56^, ^59^ Participants described how they felt about seeking help.^32^, ^33^, ^34^, ^35^, ^37^, ^41^, ^42^, ^43^, ^44^, ^46^, ^47^, ^48^, ^49^, ^50^, ^51^, ^52^, ^53^, ^54^, ^55^, ^56^, ^57^, ^58^, ^59^, ^61^, ^62^, ^63^ Support, particularly from family and friends, was widely regarded as having positive effects in relation to self‐management. However, there was also a sense of dependency and being a burden.

#### 3.3.3 Social

This dimension captures social roles and behaviour (eg interaction with others). Many studies described challenges and disruptions to domestic roles.^31^, ^32^, ^34^, ^35^, ^41^, ^42^, ^43^, ^44^, ^45^, ^46^, ^47^, ^49^, ^50^, ^51^, ^52^, ^53^, ^54^, ^55^, ^56^, ^57^, ^58^, ^59^, ^61^, ^62^ A small number of studies explored gendered dimensions of managing RA.^43^, ^45^, ^50^, ^54^, ^55^, ^60^, ^61^ Some men reported that relying on others to help with domestic tasks, such as chopping wood or DIY, could undermine their sense of masculinity. For mothers, maintaining caring roles could be physically and emotionally challenging: *‘I could not tie his [child's] little boots and had to call a neighbour, this was a trauma… I could not take my baby in my arms’*.^51^ ^(p7)^ Difficulties in maintaining a traditional, masculine role around fatherhood were also expressed: ‘*Sons look at [their] dads as being just like, “Dad, you're invincible”, and then all of sudden you can't do it’*.^43^ ^(p333)^

The challenge of maintaining employment was described.^30^, ^32^, ^33^, ^34^, ^37^, ^41^, ^43^, ^44^, ^50^, ^51^, ^52^, ^53^, ^54^, ^55^, ^57^, ^58^, ^59^, ^62^, ^63^, ^64^ People reflected on how the illness affected their ability to work and how a loss of employment impacted their self‐identity. Taking sick leave was often avoided if it was felt that colleagues or employers did not recognize their illness as legitimate, or if the participant did not wish to reveal their illness for fear they would be perceived as less productive or competent. Conversely, having a supportive employer and colleagues was key to sustaining employment. Economic aspects of living with and managing RA were described in some studies.^32^, ^35^, ^41^, ^43^, ^54^, ^55^, ^56^, ^59^, ^64^ This often related to a loss of income from employment and increased medical bills. Limited means could lead to reduced participation in social and physical activities.

RA was experienced as an invisible illness ^32^, ^37^, ^41^, ^42^, ^43^, ^46^, ^50^, ^51^, ^53^, ^55^, ^57^, ^58^, ^59^, ^62^, ^64^ whereby people struggled to convince family, friends and colleagues that they were legitimately ‘ill’. Yet, the support of others was described as an essential part of self‐managing RA. Paradoxically, some participants reported a desire to conceal—or mask—their illness. RA affected personal and social relationships.^31^, ^32^, ^33^, ^34^, ^35^, ^36^, ^37^, ^41^, ^42^, ^43^, ^44^, ^45^, ^46^, ^48^, ^49^, ^51^, ^52^, ^53^, ^54^, ^55^, ^56^, ^57^, ^58^, ^59^, ^60^, ^61^, ^62^ For some, this meant depending more on family and friends for emotional or physical support. Where participants felt unsupported by family, friends or colleagues, it usually concerned a lack of emotional support: ‘*My family are not what I wished them to be. I wish they accepted me the way I am and understood my special condition’*.^56^ ^(p246)^ As Ostlund surmises ‘living with RA not only includes the person affected, but also those close to them’.^60^ ^(p254)^ Loss, loneliness and social isolation featured in people's experience, particularly during a flare with decreased participation in social outings and activities reported.^32^, ^34^, ^35^, ^36^, ^37^, ^41^, ^43^, ^44^, ^49^, ^50^, ^51^, ^52^, ^53^, ^54^, ^56^, ^57^, ^58^, ^59^, ^60^, ^62^

#### 3.3.4 Environmental

The environment refers to the setting in which people manage RA, including the built environment and climate. People with RA reflected on the accessibility of their environment.^31^, ^36^, ^44^, ^54^, ^55^, ^56^, ^58^, ^59^, ^63^ Stairs or public transport could be particularly challenging: *‘I [want to] catch the MRT [public transport system], the door's about to close but I can't hurry up. I tell myself I need to hurry to that door, but my movement just can't get me there!*’.^59^ ^(p382)^ Difficulties driving or using public transport could impact on the person's ability to attend personal and medical appointments. Weather was described in a small number of studies.^41^, ^47^, ^51^, ^56^, ^60^, ^63^ Exposure to extreme hot or cold temperatures was thought to aggregate symptoms.

#### 3.3.5 Physical

The physical symptoms described across the studies related to pain,^34^, ^37^, ^41^, ^42^, ^44^, ^46^, ^47^, ^49^, ^51^, ^53^, ^54^, ^55^, ^56^, ^57^, ^58^, ^59^, ^60^ fatigue,^41^, ^44^, ^47^, ^51^, ^52^, ^53^, ^56^, ^60^ reduced mobility and strength,^41^, ^44^, ^46^, ^47^, ^48^, ^49^, ^51^, ^53^, ^58^, ^60^, ^63^ and swelling and inflammation.^37^, ^41^, ^47^, ^53^ The pain associated with RA can be particularly unbearable, as one woman describes: *‘I can't do anything to get release from severe pain except crying’*.^46^ ^(p242)^ Thus, individuals reconsidered how to relate to the **body** as ill, disabled or deformed.^30^, ^32^, ^37^, ^41^, ^44^, ^46^, ^49^, ^50^, ^51^, ^53^, ^54^, ^55^, ^57^, ^58^, ^59^, ^60^, ^62^, ^64^ Learning to listen to the body was recognized as an important tool of self‐management.

#### 3.3.6 Technological

Participants identified various technologies they used to manage RA. Technologies refer to the tools that people used to self‐manage their illness, including products such as medical devices and medications, as well as services provided by health‐care professionals. Assistive devices and aids were often used.^31^, ^36^, ^41^, ^43^, ^47^, ^50^, ^54^, ^56^, ^62^, ^63^, ^64^ Generally, they were perceived as opportunities for greater independence. Some studies described the experience of taking medication, including alternative medicines and treatments.^31^, ^32^, ^33^, ^35^, ^36^, ^37^, ^41^, ^42^, ^43^, ^44^, ^46^, ^47^, ^49^, ^50^, ^51^, ^55^, ^56^, ^57^, ^58^, ^59^, ^60^ Medication was often experienced in relation to time, the long and painful wait for a new medication to work or a flare to pass, and anxieties around how long a treatment will continue to be effective. Experiences of health services were discussed in relation to interactions with health‐care professionals (HCP).^31^, ^32^, ^33^, ^36^, ^41^, ^42^, ^43^, ^44^, ^45^, ^46^, ^47^, ^51^, ^52^, ^53^, ^55^, ^56^, ^57^, ^58^, ^59^, ^60^, ^61^, ^62^ Dissatisfaction was expressed in relation to long waiting times, feeling rushed at appointments, lack of information and perceived disinterest from HCPs. However, where a positive relationship with the HCP had developed this could provide enormous support.

## 4 DISCUSSION

We systematically reviewed and synthesized 32 peer‐reviewed qualitative studies to understand (a) how patients experience the self‐management of RA and (b) the aspects of self‐management they describe as most pertinent. Our findings assert that people with RA experience self‐management across six domains: cognitive‐emotional, behavioural, social, environmental, physical and technological. Analysis suggests that cognitive‐emotional and social domains are most pertinent to living with RA. In particular, renegotiating dimensions of ‘the self’ is an important part of the cognitive‐emotional work of self‐management and illustrates the lived experience of RA in relation to the person's self‐confidence, self‐esteem and self‐worth; self‐efficacy and self‐empowerment; and self‐identity and self‐perception of the illness. These aspects of the self are challenged at various points in the disease trajectory; for example, self‐identify may be disrupted in the context of lost employment,^58^ or self‐confidence may be reduced when the person compares their current and past functioning or participation.^46^ This is consistent with the sociological view of chronic illness as biologic disruption, a disruptive event which prompts a rethinking of the self.^65^, ^66^ Managing a shift in self‐identity is reported in other long‐term chronic conditions, including stroke,^67^ Parkinson's disease,^68^ multiple sclerosis ^69^ and other forms of arthritis ^70^ and multi‐morbidity.^71^ These studies similarly identify social, emotional and behavioural themes (such as hope, adjustment, spirituality), suggesting universal features of the lived experience of self‐managing chronic illnesses. Further, comparative research should explore the emphasis and prioritization of these themes in the self‐management of chronic illness.

Psychological factors have long been recognized as contributing to the aetiology and course of serious illnesses, such as RA. Alfred Mueller and colleagues identified and discussed psychological and social factors, especially personality traits, in relation to rheumatoid arthritis in the 1950s and 1960s.^72^ This analysis finds that self‐management is rooted in the cognitive ‘thinking work’ of the self. It purports that cognitive and emotional aspects of self‐management are central in adjusting to chronic illness and should be re‐examined in the context of self‐management support. Traditionally, the psychosocial aspects of self‐managing chronic and invisible illnesses, such as RA, have been considered with a medical lens ^73^ with the design of self‐management supports derived from biomedical perspectives, and often focused on clinical engagement with HCPs and health‐care networks.^8^, ^13^ Indeed, in this synthesis the prevalence of clinical fieldwork settings and medical and nursing affiliations of first authors suggests the continued dominance of medical perspectives.

Much attention and resources have been placed upon the ‘doing’ of self‐management, particularly behaviour change.^74^ However, there has been some evidence to suggest increased success in behaviour change where interventions have focused on cognitive and emotional aspects, such as positive self‐affirmation and self‐confidence.^14^, ^75^ This review adds further weight to that argument and suggests greater attention should be placed upon supporting people with RA to negotiate cognitive and emotional disruptions—specifically with regard to self‐concept, self‐efficacy and self‐esteem. Our analysis highlights the integral role of social networks and resources, especially familial support. Indeed, programmes that train family in supportive communication techniques can improve patient symptom management and health behaviours.^76^ However, perceptions of support may be linked to the individual's sense of self‐efficacy or personal and social competence,^77^ thus addressing concepts related to the self is a fundamental step.

Qualitative evidence on the self‐management of RA has typically been generated from standard qualitative methodologies, such as semi‐structured interviews. We observe a lack of participatory techniques to understand the lived experience of self‐managing RA. While advances are being made in the development of self‐management interventions, particularly technological,^78^, ^79^, ^80^ it is vital that these interventions are informed by a deep understanding of the lived experience of the patient community and their needs and concerns. We suggest the incorporation of techniques that emphasize participation, such as participatory action research, to deepen knowledge of the dimensions of self‐concept, self‐esteem and self‐efficacy.

### 4.1 Strengths and limitations

This is the first meta‐synthesis of the lived experience of self‐managing rheumatoid arthritis. As such, the findings will be of interest to health researchers, patient and public involvement and engagement (PPIE) partners, clinicians, patients and policymakers in the area of chronic disease self‐management. There are, however, a number of limitations that should be taken into consideration. The systematic review process generated more studies than expected. It was therefore decided that the original study design of meta‐ethnography would be unmanageable. On reflection, using a structured approach to selecting an appropriate qualitative evidence synthesis approach would have been beneficial.^64^

We maximized the rigour of study selection through team consensus, development of a standardized screening instrument (Appendix B) and independent double screening of citations with a purposive sample of full‐text articles. Within our relatively small team, we prioritized team input into robust data analysis. As a result, the data extraction process and most full‐text articles were single‐screened. This has implications for search comprehensiveness and increases the chance of missing relevant studies that would otherwise be eligible for inclusion. Additionally, although limiting our search to peer‐reviewed journal articles might increase the methodological quality and reporting of the included studies, it is possible that we overlooked rich, relevant experiences and insights from people with arthritis from non–peer‐reviewed sources, including grey literature, reports, Internet blog posts and social media. Similarly, we excluded non‐English language articles reflecting a language bias. Thus, we are aware the literature in this review is skewed towards academic and professional frames of patient experiences. While we did not seek additional involvement of external stakeholders, due to time and resource limitations, the team brought lived experience of arthritis [TK], [MM] and self‐managing chronic illness [SD] enhancing reflexivity of the patient perspective.^65^

We have presented a comprehensive overview of self‐management strategies reported by people with RA; however, their efficacy is not assessed. Future research could evaluate whether the self‐management strategies described here lead to improved health outcomes, particularly across social dimensions of gender, class and race.

## 5 CONCLUSIONS

This systematic review and meta‐synthesis of qualitative evidence on the lived experience of RA articulates the experience of self‐management and points to its many and diverse dimensions. It helpfully conceptualizes a number of domains in which self‐management is experienced. It provides a comprehensive and up‐to‐date summary of the self‐management of RA and crucially explores how these dimensions feature in people's lived experience. Our findings highlight the diverse challenges of self‐managing RA, including adapting to the illness, balancing roles, pacing activities, feelings of burden, struggles with feelings of dependency and acceptance, and strategies to overcome these challenges identified by people with RA. Crucially, it emphasizes the importance of negotiating dimensions of the self (self‐concept, self‐esteem and self‐efficacy) in supporting the self‐management of RA.

## CONFLICT OF INTEREST

The authors declare that there is no conflict of interest.

## Data Availability

The data that support the findings of this study are available from the corresponding author upon reasonable request.

## APPENDIX A

### Search strategy

**Table:**

Thesaurus terms						Keywords free text
	EMBASE	MEDLINE	CINAHL	PsycINFO	ASSIA	
S	'rheumatoid arthritis'/exp	"Arthritis, Rheumatoid"[Mesh]	(MH "Arthritis, Rheumatoid+") OR (“Rheumatoid Arthritis” OR “arthritis, rheumatoid”)	MAINSUBJECT.EXACT.EXPLODE("Rheumatoid Arthritis") OR “Rheumatoid Arthritis” OR “arthritis, rheumatoid”	MAINSUBJECT.EXACT.EXPLODE("Rheumatoid arthritis") OR “Rheumatoid Arthritis” OR “arthritis, rheumatoid”	“Rheumatoid Arthritis” OR “arthritis, rheumatoid”
PI	'self care'/exp OR 'self medication'/exp OR 'disease management'/exp OR 'coping behavior'/exp OR 'self concept'/exp OR 'lifestyle and related phenomena'/exp OR 'quality of life'/exp OR 'cost of illness'/exp OR 'disease burden'/exp	"Self‐Management"[Mesh:noexp] OR "Self Care"[Mesh] OR "Disease Management"[Mesh] OR "Adaptation, Psychological"[Mesh] OR "Self Concept"[Mesh] OR "Self Efficacy"[Mesh] OR "Internal‐External Control"[Mesh] OR "Self‐Control"[Mesh] OR "Resilience, Psychological"[Mesh]	(MM "Self Care") OR (MH "Self Concept+") OR (MH "Support Groups") OR (MH "Self Administration") OR (MH "Self Care Agency") OR (MH "Coping+") OR (MH "Disease Management+") OR (MH "Adaptation, Psychological+") OR (MH "Behavior and Behavior Mechanisms+") OR (MH "Control (Psychology)+")	MAINSUBJECT.EXACT.EXPLODE("Self‐Management") OR MAINSUBJECT.EXACT.EXPLODE("Self‐Care Skills") OR MAINSUBJECT.EXACT.EXPLODE("Self‐Help Techniques") OR MAINSUBJECT.EXACT.EXPLODE("Self‐Concept") OR MAINSUBJECT.EXACT.EXPLODE("Self‐Medication") OR MAINSUBJECT.EXACT.EXPLODE("Coping Behavior") OR MAINSUBJECT.EXACT.EXPLODE("Self‐Efficacy") OR MAINSUBJECT.EXACT.EXPLODE("Self‐Actualization") OR MAINSUBJECT.EXACT.EXPLODE("Empowerment") OR MAINSUBJECT.EXACT.EXPLODE("Self‐Control")	MAINSUBJECT.EXACT.EXPLODE("Selfmanagement") OR MAINSUBJECT.EXACT.EXPLODE("Selfcare") OR MAINSUBJECT.EXACT.EXPLODE("Selfmedication") OR MAINSUBJECT.EXACT.EXPLODE("Selfhelp") OR MAINSUBJECT.EXACT.EXPLODE("Selfconcept") OR MAINSUBJECT.EXACT.EXPLODE("Disease management") OR MAINSUBJECT.EXACT("Adaptation") OR MAINSUBJECT.EXACT.EXPLODE("Selfefficacy") OR MAINSUBJECT.EXACT.EXPLODE("Selfactualization") OR MAINSUBJECT.EXACT.EXPLODE("Selfempowerment") OR MAINSUBJECT.EXACT.EXPLODE("Locus of control") OR MAINSUBJECT.EXACT.EXPLODE("Selfregulation") OR MAINSUBJECT.EXACT.EXPLODE("Resilience") OR MAINSUBJECT.EXACT.EXPLODE("Coping skills") OR MAINSUBJECT.EXACT.EXPLODE("Coping style") OR MAINSUBJECT.EXACT.EXPLODE("Coping") OR MAINSUBJECT.EXACT.EXPLODE("Coping strategies")	“Self Management” OR Self‐management OR Selfmanagement OR “Self treatment” OR self‐treatment OR selftreatment OR “self care” OR self‐care OR selfcare OR “self help” OR self‐help OR “self medication” OR self‐medication OR “Disease management” OR “Disease Managements” OR impact* OR adapt* OR cope* OR coping OR behaviour* OR behavior* OR actualiz* OR self‐efficacy OR selfefficacy OR “self efficacy” OR empower* OR control OR regulat* OR resilience OR perspective OR perception OR attitude OR cost OR “treatment burden” OR “Disease burden”
D	'semi structured interview'/exp OR 'focus group'/exp OR 'participant observation'/exp OR 'non participant observation'/exp OR 'case study'/exp OR 'field study'/exp OR 'audio recording'/exp OR 'grounded theory'/exp OR 'phenomenology'/exp OR 'ethnography'/exp OR 'participatory research'/exp OR 'action research'/exp OR 'photovoice'/exp OR 'thematic analysis'/exp OR 'content analysis'/exp OR 'life history'/exp	"Focus Groups"[Mesh] OR "Grounded Theory"[Mesh] OR "Anthropology, Cultural"[Mesh] OR "Community‐Based Participatory Research"[Mesh] OR "Life Change Events"[Mesh]	(MH "Interviews+") OR (MH "Observational Methods+") OR (MH "Self Report+") OR (MH "Narratives") OR (MH "Delphi Technique") OR (MH "Focus Groups") OR (MH "Audiorecording") OR (MH "Case Studies") OR (MH "Ethnographic Research") OR (MH "Grounded Theory") OR (MH "Phenomenological Research") OR (MH "Grounded Theory") OR (MH "Action Research") OR (MH "Thematic Analysis") OR (MH "Content Analysis") OR (MH "Life Histories")	MAINSUBJECT.EXACT.EXPLODE("Observation Methods") OR MAINSUBJECT.EXACT.EXPLODE("Self‐Report") OR MAINSUBJECT.EXACT.EXPLODE("Content Analysis") OR MAINSUBJECT.EXACT.EXPLODE("Grounded Theory") OR MAINSUBJECT.EXACT.EXPLODE("Phenomenology") OR MAINSUBJECT.EXACT("Ethnography") OR MAINSUBJECT.EXACT("Action Research")	MAINSUBJECT.EXACT.EXPLODE("Interviews") OR MAINSUBJECT.EXACT.EXPLODE("Focus groups") OR MAINSUBJECT.EXACT.EXPLODE("Participant observation") OR MAINSUBJECT.EXACT("Tape recordings") OR MAINSUBJECT.EXACT.EXPLODE("Ethnography") OR MAINSUBJECT.EXACT.EXPLODE("Phenomenology") OR MAINSUBJECT.EXACT.EXPLODE("Participatory action research") OR MAINSUBJECT.EXACT.EXPLODE("Content analysis") OR MAINSUBJECT.EXACT.EXPLODE("Grounded theory") OR MAINSUBJECT.EXACT.EXPLODE("Life histories")	semistructured OR semi‐structured OR "semi structured" OR “unstructured interview” OR “unstructured interviews” OR “focus group” OR “participant observation” OR “non‐participant observation” OR “case study” OR “case studies” OR “field study” OR “field studies” OR “audio recording” OR audiorecording OR “grounded theory” OR phenomenolog* OR ethnograph* OR metaethnography OR meta‐ethnography OR “participatory research” OR “community‐based participatory research” OR “action research” OR “participatory action research” OR photovoice OR “photo voice” OR “thematic analysis” OR “content analysis” OR “life history” OR “Life histories”
E	'personal experience'/exp OR 'patient experience'/exp	"Personal Narratives as Topic"[Mesh] OR "Personal Satisfaction"[Mesh]	(MH "Life Experiences+")	MAINSUBJECT.EXACT.EXPLODE("Life Experiences")	MAINSUBJECT.EXACT.EXPLODE("Life experiences") OR MAINSUBJECT.EXACT.EXPLODE("Personal experiences") OR	“Lived experience” OR “Personal experience” OR “patient experience” OR “life experience” OR experience
R	'qualitative research'/exp OR 'mixed methods'/exp	"Qualitative Research"[Mesh]	(MH "Qualitative Studies")	MAINSUBJECT.EXACT.EXPLODE("Qualitative Research")	MAINSUBJECT.EXACT.EXPLODE("Qualitative data") OR MAINSUBJECT.EXACT.EXPLODE("Qualitative methods") OR MAINSUBJECT.EXACT.EXPLODE("Qualitative research") OR MAINSUBJECT.EXACT.EXPLODE("Qualitative analysis")	“qualitative research” OR “qualitative studies” OR “qualitative study” OR “mixed method” OR “mixed methods”

## APPENDIX B

### Inclusion/exclusion criteria

**Table:**

Inclusion criteria	
Sample	Adults aged 18+ years who have a diagnosis of rheumatoid arthritis.
	No restrictions will be applied based on severity or duration of illness or any other demographic variable.
Phenomenon of interest	Perceptions, attitudes, perspectives and experiences of adults with RA towards: The lived experience of RA Self‐management, disease management, self‐treatment, self‐medication Self‐care, self‐help, self‐concept, self‐efficacy, self‐actualization, self‐empowerment Resilience, coping, psychological adaptation Cost of illness, treatment burden
Design, evaluation, research type	Studies in the English language, from any publication date and any geographical location.
	Studies that address the lived experience of self‐management or self‐care or coping in rheumatoid arthritis patients.
	Studies conducted using qualitative methods (eg interviews, focus groups, ethnography). This includes: Primary and secondary qualitative studies. A qualitative study as part of a mixed‐methods study. Mixed‐method studies are included (only when qualitative data were reported separately)
	Peer‐reviewed journal articles only.
	Studies involving people with diseases other than RA (ie mixed populations) will be included only if outcomes/results for people with RA are presented separately, and only if this is suggested within the title or abstract.

**Table:**

Exclusion Criteria	
Sample	Children (<18 years)
	No diagnosis of RA
Phenomenon of interest	Studies that are not qualitative in nature
	Do not focus on lived experience or address the phenomenon of interest, that is self‐management or self‐care.
	Do not focus on the person with RA specifically (eg experience of spouse; experience of health‐care provider/ clinician).
	Do not address the disease area of interest specifically or are ambiguous, that is juvenile arthritis, including JIA and JRA, chronic inflammatory arthritis, chronic illness or disease, chronic pain, chronic fatigue, etc
Design, evaluation, research type	Any perspectives other than people with RA.
	Any quantitative study (protocols, testing measures, RCT, non‐RCT, clinical studies, for example drug efficacy, observational, cohort, case control).
	Studies that do not state or describe a qualitative method of data analysis.
	Non‐empirical articles including opinion pieces, commentaries, theoretical articles and treatment guideline documents.
	Personal accounts that do not employ qualitative methods of data collection and analysis.
	Grey literature/ non–peer‐reviewed works, including book chapters, policy reports, technical reports, short reports, conference proceedings and reports, dissertations, commentaries/ editorials.
	Dissertations/theses/conference proceedings/published abstracts—however, attempts will be made to source full‐length articles if abstracts are otherwise eligible.
	Secondary studies (ie systematic reviews, meta‐analysis, meta‐ethnography, qualitative evidence syntheses).

## APPENDIX C

### CASP scores ^*^ A score of 1 indicated that the item had been thoroughly discussed in the manuscript; a score of 0.5 meant it was unclear or there was limited discussion of the item; and a score of 0 indicated that the item was not discussed or was inadequate to the research design. Overall scores >9 were deemed ; scores between 7 and 9 were deemed ; and scores <7 were low quality.

**Table:**

Article	Bala et al (2017)	Bergsten et al (2011)	Brown et al (1995)	Chaleshgar‐Kordasiabi et al (2018)	Coty et al (2013)	Flurey et al (2014)	Flurey et al (2017)	Gronning et al (2011)	Hooper et al (2004)	Hwang et al (2004)	Kett et al (2010)	Kristiansen et al (2012b)	Kristiansen et al (2012a)	Laquinta et al (2004)	Lempp et al (2006)	Li Wen et al (2017)
Q1 Was there a clear statement of the aims of the research?	1	1	1	1	1	1	1	1	1	1	1	1	1	1	1	1
Q2 Is a qualitative methodology appropriate?	1	1	1	1	1	1	1	1	1	1	1	1	1	1	1	1
Q3 Was the research design appropriate to address the aims of the research?	1	1	1	1	1	1	1	1	1	1	1	1	1	1	1	1
Q4 Was the recruitment strategy appropriate to the aims of the research?	1	1	1	1	0.5	1	1	1	0.5	1	1	1	1	0.5	1	1
Q5 Was the data collected in a way that addressed the research issue?	1	1	1	1	0	1	1	1	1	1	1	1	1	1	1	1
Q6 Has the relationship between researcher and participants been adequately considered?	1	0	1	0	0	0	1	1	0	0	1	0	0	1	0	1
Q7 Have ethical issues been taken into consideration?	1	1	1	1	0.5	0.5	0.5	0.5	0.5	1	0.5	0.5	0.5	0.5	0.5	1
Q8 Was the data analysis sufficiently rigorous?	1	1	0.5	1	1	1	1	1	0.5	1	1	1	1	1	1	1
Q9 Is there a clear statement of findings?	1	1	1	1	1	1	1	1	1	1	1	1	1	1	1	1
Q10 How valuable is the research?	1	0.5	0.5	0.5	1	0.5	0.5	1	0.5	1	1	1	0.5	1	1	1
**Score**	**10**	**8.5**	**9**	**8.5**	**7**	**8**	**9**	**9.5**	**7**	**9**	**9.5**	**8.5**	**8**	**9**	**8.5**	**10**

**Table:**

CASP scores *(table continued)*																
Article	Lutze et al (2007)	Malm et al (2017)	Malm et al (2016)	Melanson et al (1995)	Melanson et al (2003)	Mitton et al (2007)	Ostlund et al (2015)	Ostlund et al (2016)	Ottenvall Hammar et al (2013)	Pedraz‐Marcos et al (2018)	Prodinger et al (2014)	Ryan (1996)	Shaul (1995)	Stamm et al (2008)	Stenstrom et al (1993)	Yoshida et al (2004)
Q1 Was there a clear statement of the aims of the research?	1	1	1	1	1	1	1	1	1	1	1	1	1	1	1	1
Q2 Is a qualitative methodology appropriate?	1	1	1	1	1	1	1	1	1	1	1	1	1	1	1	1
Q3 Was the research design appropriate to address the aims of the research?	1	1	1	1	1	1	0.5	1	1	1	1	0.5	0.5	1	1	0.5
Q4 Was the recruitment strategy appropriate to the aims of the research?	1	1	1	1	1	1	1	1	1	1	1	0.5	0.5	1	1	0.5
Q5 Was the data collected in a way that addressed the research issue?	1	1	1	1	1	1	1	1	1	1	1	1	1	1	1	1
Q6 Has the relationship between researcher and participants been adequately considered?	0	0.5	0.5	0	0	1	0.5	1	1	1	0.5	1	0	1	0	0
Q7 Have ethical issues been taken into consideration?	0	1	1	1	0.5	1	0.5	1	1	1	0.5	0.5	0.5	0.5	0.5	0.5
Q8 Was the data analysis sufficiently rigorous?	0.5	1	1	1	1	1	1	1	1	1	1	1	1	1	1	1
Q9 Is there a clear statement of findings?	1	1	1	1	1	1	1	1	1	1	1	1	1	1	1	1
Q10 How valuable is the research?	0.5	1	1	1	0	1	1	1	1	1	0.5	1	1	1	1	1
**Score**	**7**	**9.5**	**9.5**	**9**	**10**	**10**	**8.5**	**10**	**10**	**10**	**8.5**	**8**	**7.5**	**9.5**	**8.5**	**7.5**

### Summary of CASP scores

**Table:**

Number of Answers Across All Included Studies				
	CASP question	Yes	Unclear	No
	*RIGOUR—Are the results valid?*			
Q1	Was there a clear statement of the aims of the research?	32	0	0
Q2	Is a qualitative methodology appropriate?	32	0	0
Q3	Was the research design appropriate to address the aims of the research?	28	4	0
Q4	Was the recruitment strategy appropriate to the aims of the research?	26	6	0
Q5	Was the data collected in a way that addressed the research issue?	30	1	1
Q6	Has the relationship between researcher and participants been adequately considered?	14	3	15
	*CREDIBILITY—What are the results?*			
Q7	Have ethical issues been taken into consideration?	15	16	1
Q8	Was the data analysis sufficiently rigorous?	30	2	0
Q9	Is there a clear statement of findings?	32	0	0
	*RELEVANCE—Will the results help locally?*			
Q10	How valuable is the research?	24	7	1

## APPENDIX D

### Characteristics of included studies

**Table:**

	First author	Year	Journal	Disciplinary affiliation	Country	Sampling	Sample size	Disease	Data collection^a^	Location^b^	Research design/methodology
1	Bala, S.	2017	J Clin Nurs	Health Sciences	Sweden	Strategic	10	RA	SSI	Mix of non/clinical	Hermeneutic phenomenological
2	Bergsten, U.	2011	Open Nursing Journal	Health Sciences	Sweden	Purposeful	16	RA	SSI	Clinical	Grounded/ explorative
3	Brown, S.	1995	Journal of Advanced Nursing	Nursing	UK	Judgement	7	RA	Non‐directive conversational interviews	Unspecified	Qualitative, narrative analysis
4	Chaleshgar‐Kordasiabi, M.	2018	Musculoskeletal Care	Public Health	Iran	Purposeful	30	RA	SSI	Clinical	Content analysis
5	Coty, M.	2013	Journal of Research in Nursing	Nursing	USA	Unspecified (drawn from larger study)	16	RA	SSI	Telephone	Thematic analysis
6	Flurey, C. A.	2017	Arthritis Care & Research	Health and Applied Sciences	UK	Unspecified (possibly purposive)	22	RA	Focus groups	Unspecified	Thematic analysis
7	Flurey, C. A.	2014	Rheumatology	Public Health/Health Psychology	UK	Purposeful	15	RA	SSI	Clinical	Thematic analysis
8	Gronning, K.	2011	Clin Rheumatol	Public Health	Norway	Patients already enrolled in an RCT	26	Mixed, RA (18)	SSI	Clinical	Systematic text condensation
9	Hooper, H.	2004	Musculoskeletal Care	Primary Care Sciences Research	UK	Unspecified (drawn from RCT)	10	RA	SSI	Clinical	Thematic analysis
10	Hwang, EJ	2004	International Journal of Nursing Studies	Unknown (Nursing?)	South Korea	Theoretical	5	RA	Unstructured interviews	Non‐clinical	Phenomenology
11	Kett, C.	2010	Musculoskeletal Care	Rheumatology, Medicine	UK	Purposeful	21	RA	SSI	Non‐clinical	Grounded
12	Kristiansen, T. M.	2012b	Musculoskeletal Care	Health Sciences	Denmark	Drawn from RCT	11	RA	Focus groups (x2)	Clinical site	Content analysis
13	Kristiansen, T. M.	2012a	Musculoskeletal Care	Health Sciences	Denmark	Purposeful	32	RA	Focus groups (x6)	Clinical site	Content analysis
14	Laquinta, M. L.	2004	Journal of Nursing Care Quality	Nursing	USA	Purposeful	6	RA	SSI	Non‐clinical	Descriptive
15	Lempp, H.	2006	Chronic Illness	Rheumatology, Medicine	UK	Quota	26	RA	SSI	Mix of non/clinical	Thematic analysis
16	Li Wen, Poh	2017	Clinical Nursing Research	Nursing	Singapore	Purposeful	16	RA	SSI	Unspecified	Descriptive, thematic analysis
17	Lutze, U.	2007	Scand J Caring Sci	Occupational therapy	Sweden	Random selection from BARFOT study	23	RA	Focus groups (x4)	Unspecified	Unspecified
18	Malm, K.	2017	SAGE Open Med	Rheumatology, Medicine	Sweden	Strategic sampling from BARFOT study	22	RA	SSI	Clinical site	Phenomenology
19	Malm, K.	2016	International journal of qualitative studies on health and well‐being	Rheumatology, Medicine	Sweden	Strategic	22	RA	SSI	Non‐clinical	Descriptive and explorative design, content analysis
20	Melanson, P. M.	1995	Activities, Adaptation & Aging	Nursing	Canada	Stratified, convenience	77	RA	SSI	Non‐clinical	Phenomenology, content analysis
21	Melanson, PM	2003	Journal of Advanced Nursing	Nursing	Canada	Convenience	48	RA	SSI	Non‐clinical	Longitudinal, descriptive design; content analysis
22	Mitton, D. L.	2007	Musculoskeletal Care	Rheumatology, Medicine	UK	Unspecified	7	RA	SSI	Clinical site	Phenomenology
23	Ostlund, G.,	2015	Musculoskeletal Care	Social Work	Sweden	From TIRA‐2 study	45	RA	SSI	Mix of non/clinical	Phenomenology (critical incident technique), content analysis
24	Ostlund, G.	2016	Musculoskeletal Care	Social Work	Sweden	Purposeful sampling from TIRA‐2 study	25	RA	SSI	Mix of non/clinical	Content analysis
25	Ottenvall Hammar, I.	2013	Scand J Occup Ther	Physio and Occupational Therapy	Sweden	Unspecified	9	Mixed, RA (7)	SSI	Mix of non/clinical	Phenomenology
26	Pedraz‐Marcos, A.	2018	Clin Nurs Res	Nursing	Spain	Unspecified	19	RA	SSI	Mix of non/clinical	Phenomenology, thematic analysis
27	Prodinger, B.	2014	Disability and Rehabilitation: An International, Multidisciplinary Journal	Rheumatology, Medicine	Austria	Unspecified	7	RA	SSI, participant observations, texts	Unspecified	Institutional ethnography
28	Ryan, S.	1996	Nursing Standard	Nursing	UK	Unspecified	7	RA	SSI	Unspecified	Phenomenology
29	Shaul, M. P.	1995	Arthritis Care and Research	Nursing	USA	Longitudinal panel	30	RA	SSI	Unspecified	Constant comparative analytic process; content analysis; naturalistic inquiry
30	Stamm, T.	2008	Qualitative Health Research	Rheumatology, Medicine	Austria	Purposeful (maximum variation sampling)	10	RA	SSI x 2‐3 times p.p.	Mix of non/clinical	Narrative biographic methodology; constant comparative content analysis
31	Stenstrom, C. H.	1993	Physiotherapy Theory & Practice	Physical Therapy	Sweden	Unspecified	9	RA	SSI	Unspecified	Phenomenology
32	Yoshida, K.	2004	Physiotherapy Theory & Practice	Physical Therapy and Rehab (Medicine)	Canada	Convenience	46	RA	SSI	Non‐clinical	Grounded

^a^Semi‐structured interviews (SSI).

^b^Non‐clinical setting refers to participant's home, work or place of their convenience or community health‐care setting, or researcher's office. Clinical setting refers to private room or otherwise in hospital or outpatient clinic.

## APPENDIX E

### Enhancing transparency in reporting the synthesis of qualitative research: the ENTREQ statement

**Table:**

No	Item	Guide and description	Section	Page
1	Aim	State the research question the synthesis addresses.	Introduction	2
2	Synthesis methodology	Identify the synthesis methodology or theoretical framework which underpins the synthesis, and describe the rationale for choice of methodology *(eg meta‐ethnography, thematic synthesis, critical interpretive synthesis, grounded theory synthesis, realist synthesis, meta‐aggregation, meta‐study, framework synthesis)*.	Data synthesis	3
3	Approach to searching	Indicate whether the search was pre‐planned (*comprehensive search strategies to seek all available studies)* or iterative (*to seek all available concepts until the theoretical saturation is achieved)*.	Methods and search strategy	2‐3
4	Inclusion criteria	Specify the inclusion/exclusion criteria *(eg, in terms of population, language, year limits, type of publication, study type)*.	Search strategy	2‐3
5	Data sources	Describe the information sources used (eg, *electronic databases (MEDLINE, EMBASE, CINAHL, PsycINFO, EconLit), grey literature databases (digital thesis, policy reports), relevant organizational websites, experts, information specialists, generic web searches (Google Scholar) hand searching, reference lists)* and when the searches conducted, provide the rationale for using the data sources.	Search strategy	2‐3
6	Electronic Search strategy	Describe the literature search *(eg, provide electronic search strategies with population terms, clinical or health topic terms, experiential or social phenomena related terms, filters for qualitative research and search limits)*.	Search strategy	2‐3
7	Study screening methods	Describe the process of study screening and sifting *(eg title, abstract and full‐text review, number of independent reviewers who screened studies)*.	Study selection	3
8	Study characteristics	Present the characteristics of the included studies *(eg year of publication, country, population, number of participants, data collection, methodology, analysis, research questions)*.	Characteristics of included studies and Appendix D	4
9	Study selection results	Identify the number of studies screened and provide reasons for study exclusion *(eg for comprehensive searching, provide numbers of studies screened and reasons for exclusion indicated in a figure/flow chart; for iterative searching describe reasons for study exclusion and inclusion based on modifications to the research question and/or contribution to theory development)*.	Study selection and Figure 1 PRISMA flow chart	3‐4
10	Rationale for appraisal	Describe the rationale and approach used to appraise the included studies or selected findings *(eg assessment of conduct (validity and robustness), assessment of reporting (transparency), assessment of content and utility of the findings)*.	Critical appraisal and quality of included studies	3‐5
11	Appraisal items	State the tools, frameworks and criteria used to appraise the studies or selected findings *(eg Existing tools: CASP, QARI, COREQ, Mays and Pope* ^25^ *; reviewer developed tools; describe the domains assessed: research team, study design, data analysis and interpretations, reporting)*.	Critical appraisal	3
12	Appraisal process	Indicate whether the appraisal was conducted independently by more than one reviewer and if consensus was required.	Critical appraisal	3
13	Appraisal results	Present results of the quality assessment and indicate which articles, if any, were weighted/excluded based on the assessment and give the rationale.	Quality of included studies	4‐5
14	Data extraction	Indicate which sections of the primary studies were analysed and how were the data extracted from the primary studies? *(eg all text under the headings ‘results/conclusions’ were extracted electronically and entered into a computer software)*.	Data extraction	3
15	Software	State the computer software used, if any.	Data extraction and study selection	3‐4
16	Number of reviewers	Identify who was involved in coding and analysis.	Data synthesis	3
17	Coding	Describe the process for coding of data *(eg line‐by‐line coding to search for concepts)*.	Data synthesis	3
18	Study comparison	Describe how were comparisons made within and across studies *(eg subsequent studies were coded into pre‐existing concepts, and new concepts were created when deemed necessary)*.	Data synthesis	3
19	Derivation of themes	Explain whether the process of deriving the themes or constructs was inductive or deductive.	Data synthesis	3
20	Quotations	Provide quotations from the primary studies to illustrate themes/constructs, and identify whether the quotations were participant quotations of the author's interpretation.	Synthesis and Table 1	5‐7
21	Synthesis output	Present rich, compelling and useful results that go beyond a summary of the primary studies (eg *new interpretation, models of evidence, conceptual models, analytical framework, development of a new theory or construct)*.	Discussion	8‐9
